# I am one of you! Team prototypicality as a facilitator for female leaders

**DOI:** 10.3389/fpsyg.2022.859577

**Published:** 2022-10-20

**Authors:** Alina S. Hernandez Bark, Lucas Monzani, Rolf van Dick

**Affiliations:** ^1^Department of Social Psychology, Institute of Psychology, Goethe University Frankfurt, Frankfurt, Germany; ^2^Organizational Behavior, Ivey Business School at Western University, London, ON, Canada

**Keywords:** Prototypicality, authentic leadership, social identity model of leadership, gender roles, gender stereotypes, team prototypicality

## Abstract

In the present study, we complement role congruity theory with insights from the Social Identity Model of Leadership. We propose that especially female leaders benefit from team prototypicality, i.e., being representative of the group they are leading. We assume that team prototypicality shifts the comparative frame away from higher-order categories like gender and leader roles to more concrete team-related properties and thereby reduces disadvantages for female leader that stem from the incongruity between the leader role and the female gender role stereotypes. Further, this effect should affect both (female) leaders themselves and their perception by their followers. Building on previous research, we predict, first, lower authentic leadership behavior for female than male leaders. Second, that team prototypicality positively relates to authentic leadership and trust in leader. Third, that team prototypicality has stronger relations to authentic leadership and trust in leader for female compared to male leaders. We tested assumptions in a randomized online experiment (Study 1, *N* = 315) and a cross-sectional survey study (Study 2, *N* = 300). We did not find consistent support for the assumed gender differences in authentic leadership. But our results (both in manifest and in latent analyses) show that team prototypicality—both self-perceived (Study 1) and as perceived by employees (Study 2)—is related to more authentic leadership and more trust in leader (Study 2) and that these relations are stronger for female than for male leaders. Furthermore, we tested in Study 2 an extended model including follower’s job satisfaction as the final follower outcome affected *via* team prototypicality, leader gender, authentic leadership, and trust in leader. Thereby, we found that team prototypicality has direct and indirect effects on job satisfaction as carried through authentic leadership and trust in leader, respectively. Together, the results of both studies support our assumptions and show that female leaders can reduce role incongruity barriers through high team prototypicality. Implications for future research and practical implications of these results for gender equality are discussed.

## Introduction

Women constitute almost half of today’s workforce worldwide (The World Bank, 2020), and yet, women are still under-represented in upper echelons of most FT-500 firms (Catalyst, 2021). Such under-representation has persisted for decades, even after organizations started implementing gender equality policies and quotas to help women reach and maintain leadership positions. While reasons why women struggle to emerge as and be successful leaders have been explained elsewhere (e.g., Eagly and Karau, 2002; Ryan and Haslam, 2007), these figures suggest that there is still a need for new insights that can contribute to the reduction of discrimination and prejudice women experience once they made it into a leadership position, and thus, facilitate their exercise of leadership.

From a psychological perspective, Role Congruity Theory (RCT; Eagly and Karau, 2002) captures the prevailing scholarly consensus on why women suffer a double bind and prejudice well. In short, RCT’s core proposition is that traditional gender and leader role stereotypes tend to align for men (or those who identify with the male gender), but not for women (or those who identify with the female gender). For almost two decades, empirical research has supported RCT’s claims about the mechanics of discrimination and prejudice toward women in leadership roles (Heilman, 2012; Hernandez Bark et al., 2014; Badura et al., 2018; Manzi and Heilman, 2021).

However, while RCT provides a good conceptual framework for the understanding of the mechanisms behind discrimination that female leaders are exposed to, the reports by both the World Bank and Catalyst suggest that the insights of RCT did not contribute to change the underrepresentation of women in higher leadership in contemporary firms. Thus, female leaders might benefit from additional empirical insights that can extend or complement RCT while honoring its core propositions. For example, a recent study extended RCT theory to the field of entrepreneurship, attempting to find ways to reduce the societal pressures that elicit antisocial behaviors in female leaders and entrepreneurs (Monzani et al., 2021). In the present study, we propose complementing RCT with insights from the Social Identity Model of Leadership (SIMOL; Hogg, 2001).

Following the claims of SIMOL theory, prototypicality, and especially team prototypicality, would allow female leaders to tackle some of the barriers resulting from role incongruity. Therefore, the main objective of the present study is to test if the propositions of SIMOL could complement the insights provided by Role Congruity Theory in order to support female leaders. More precisely, we inquire if prototypicality enables women to perceive themselves and be perceived by others as effective leaders (van Knippenberg et al., 2004; Van Dick and Kerschreiter, 2016). More precisely, we focus on leveraging prototypicality when leading a workgroup. Prototypicality means that the leader is perceived as “one of us,” as a model member of the team, or ideally as “the best of us” (meaning as an exemplary member of the team; Van Dick and Kerschreiter, 2016). We believe that team prototypicality, thus being representative of the team one is leading, would help female leaders overcome the obstacles and prejudice resulting from the mismatch between the female gender role stereotype (communal) and the leadership role stereotype (agentic) still prevailing in contemporary organizations. If our predictions were to be supported by our data, our work would make valuable theoretical and empirical contributions to RCT, SIMOL, and its recent extension, Identity Leadership (Haslam et al., 2021). More precisely, by shifting the focus from battling rigid societal stereotypes to managing the identity dynamics of the groups they lead, women can transcend the role incongruity described by RCT and be more effective in attaining and sustaining leadership positions, but also feel more self-expressive when occupying said positions.

**Figure 1:**
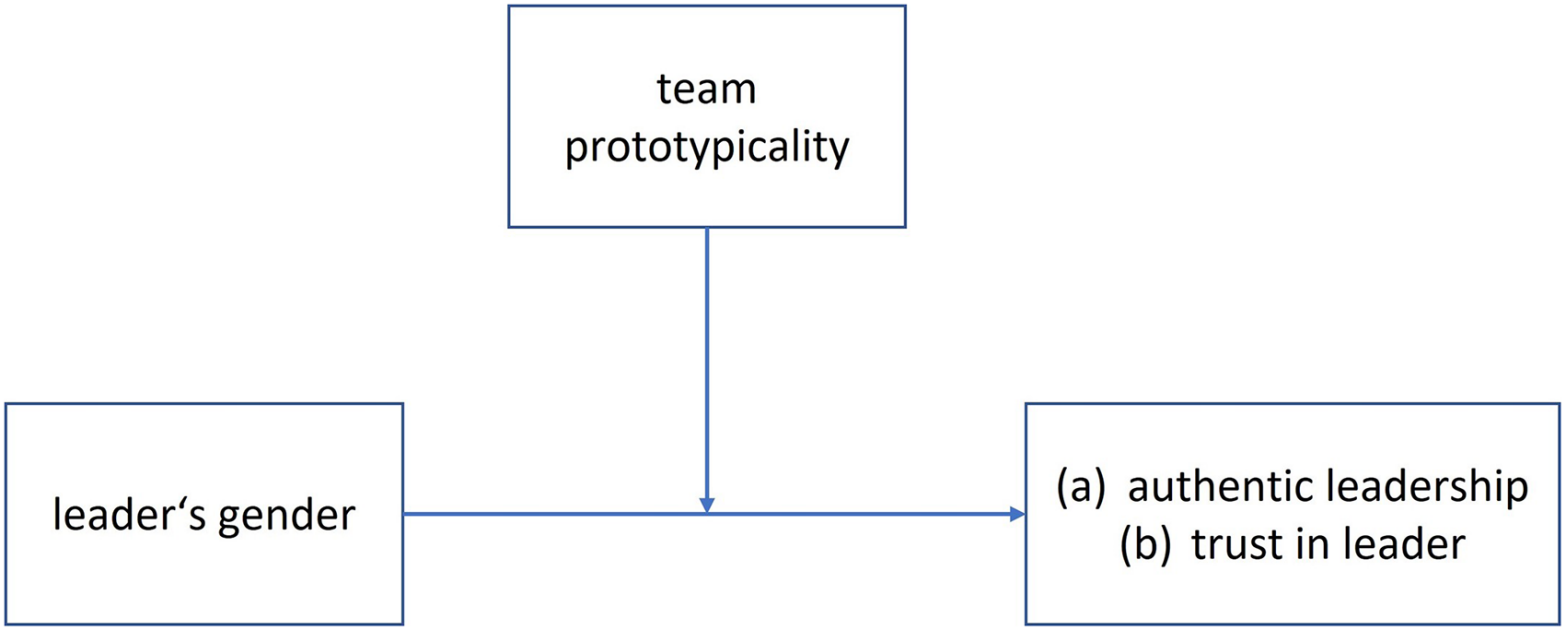
Proposed core model.

The core premise of our study is grounded on extant evidence, which suggests that leveraging a prototypical status might be a valuable tool to enhance female leadership. For example, followers of prototypical leaders tend to be more tolerant when their leaders fail to achieve goals (Giessner and van Knippenberg, 2008). Similarly, followers are also more tolerant when leaders breach existing social norms, such as ensuring procedural justice (Ullrich et al., 2009). Thus, in the present study, we propose that being perceived as “one of us in the work team,” so especially the team prototypical status, is one possibility for female leaders to overcome role incongruity issues – both for themselves and in the perception of their followers – and to increase their authentic leadership behavior as well as their employee’s trust in them. Team prototypicality focuses on both the team itself and its leader being representative for the team. We assume that such focus shifts the comparative dimension from higher-order categories like leader or gender to more concrete, team-related categories and thereby allows women to overcome problems emerging from role incongruity. Figure 1 illustrates our proposed core model.

## Theoretical framework

Leadership, understood as a social influence process, does not occur in a social vacuum, but is enacted in social groups. In organizational settings, extant theory on social categorization suggests that social groups (e.g., teams) tend to construct shared representations of what constitutes the ideal characteristics and behaviors that would describe someone belonging to said group, or what is known as a group’s *prototype* (Hogg and Terry, 2000). Unless leaders actively engage in identity entrepreneurship to (re)shape their group’s prototype (Steffens et al., 2014; van Dick et al., 2018), said prototype will likely be based on the prevailing role stereotypes of the society or culture in which the firm operates. Therefore, we theorize briefly the interplay between gender and leader roles within organizations.

### Gender and leadership

Gender stereotypes consist of descriptive, prescriptive, and proscriptive components (Eagly et al., 1995; Eagly and Karau, 2002; Cuddy et al., 2008; Rudman et al., 2012). In other words, gender stereotypes shape societal expectations on how women and men actually behave (descriptive), but also on how they should (prescriptive) and should not behave (proscriptive). Women are usually expected to display communal attributes and behaviors whereas men are usually expected to display agentic attributes and behaviors. The female gender role stereotype is associated with being concerned about the well-being of others and thereby with communal attributes such as being warm, kind, friendly, empathic, supportive, gentle, and caring (Bakan, 1966; Eagly, 1987; Williams and Best, 1990; Abele et al., 2008; Rudman et al., 2012; Hernandez Bark et al., 2014; March et al., 2016). In contrast, the male role stereotype is associated with agentic attributes such as being self-confident, ambitious, assertive, controlling, independent, dominant, and competitive (Bakan, 1966; Eagly, 1987; Williams and Best, 1990; Abele et al., 2008; Rudman et al., 2012; Hernandez Bark et al., 2014; March et al., 2016). Despite some changes, especially in the female gender stereotype, the general pattern of men being more closely associated with agentic properties and women being more closely associated with communal properties was confirmed by various research (Hentschel et al., 2019; Obioma et al., 2021) and two recent meta-analyses (Eagly et al., 2020; Hsu et al., 2021).

Schein (1973) already proposed the *think manager—think male paradigm* indicating that leader stereotypes are associated with male connotated properties. In their meta-analysis, Koenig et al. (2011) included in addition to Schein’s think manager—think male paradigm, also the *agency–communion paradigm* (Powell and Butterfield, 1979) and the *masculinity-femininity paradigm* (Shinar, 1975) to examine leader stereotype content. They found that leader stereotypes are more closely associated with males, agency, and masculinity. Thus, independent of the underlying paradigm, leadership stereotypes are more closely associated with men than with women. Although this pattern slightly decreased over time, it is still valid nowadays and to be found in recent research (Hoyt et al., 2011; Badura et al., 2018; Heilman and Caleo, 2018).

According to role theory (Biddle, 1979), conforming with social role prescriptions is one fundamental criterion for the perception and evaluation of an individual in a given social group or context, such as an organization. For example, in modern organizations, job roles describe the specific characteristics that an employee should possess to occupy such role (descriptive), and the performance expectations for those who occupy such roles (prescriptive). Similarly, most organizations have social norms and policies to address deviations from a job role prescription (proscriptive). In most organizations, managerial positions confirm with the agentic connotated “leader” role stereotype, which in turn has unfortunate implications for women in leadership roles, as RCT suggests.

RCT’s core proposition states that the traditional, prevailing gender and leader role stereotypes tend to align better for men than for women (Eagly and Karau, 2002; Heilman, 2012; Hernandez Bark et al., 2014). This incongruity between the traditional female and leader role stereotypes creates a double standard (female leaders must perform better than their male counterparts to be perceived as competent) and a double bind (to be “tough” and “nice” at the same time; Eagly and Karau, 2002). Thus, women have to overcome various obstacles on their way to leadership positions, such as perceiving managerial positions as less attractive (reduced leadership aspirations) and reduced authenticity when occupying leadership roles (Eagly, 2005; Eagly and Carli, 2007; Heilman, 2012; Hernandez Bark et al., 2014; Monzani et al., 2014).

Unfortunately, in organizational settings, gender role stereotypes tend to facilitate biases, prejudice, and discrimination against those individuals that seek roles that do not align with their stereotypical gender roles (Konrad et al., 2000; Rudman and Glick, 2001; Schein, 2001; Eagly and Karau, 2002; Eagly, 2005; Koenig et al., 2011; Heilman, 2012; Rudman et al., 2012; Hernandez Bark et al., 2014, 2016, 2021; Koch et al., 2015; Hernandez Bark et al., 2022; Junker et al., 2022). For example, regarding stereotypical biases based on gender roles, women are more closely associated with the stereotype of followers (the think follower—think female paradigm, Braun et al., 2017), and men are more closely associated with the stereotype of leaders (*think manager—think male* paradigm; Schein, 2001). Thus, women and other equity-deserving groups frequently struggle to occupy leadership roles. Despite some changes, the *think manager—think male* phenomenon still prevails. In this context, the *think manager−think male*, captures the mental picture of a typical leader containing more masculine attributes and being more strongly associated with the male gender stereotype (Schein, 2001; Koenig et al., 2011). Thus, employees operating under a female leader tend to experience cognitive dissonance due to the incongruity between the stereotypical attributes desired of women and the requirements of a leadership role. This dissonance affects both women’s self-perception and their perception by others (Heilman, 2001, 2012; Horvath and Sczesny, 2016).

The prejudice that female leaders suffer from creates substantive drawbacks for women in organizations. For example, women are ascribed less leadership potential and are evaluated less favorably in leadership positions (Heilman, 2001, 2012; Eagly and Karau, 2002; Lord and Hall, 2003). One well-known possibility for female leaders to reduce this incongruity is to embrace a transformational leadership style, as its facets include communal behaviors (e.g., showing individual consideration for their followers). However, this solution also goes along with drawbacks, as female leaders are expected to show transformational leadership behavior and thus, do not receive additional recognition when acting transformational like male leaders do (Hentschel et al., 2018). Therefore, the present study focuses on a positive leadership style that includes both communal and agentic aspects and thus, is more ambiguous regarding its fit to gender role stereotypes; precisely on authentic leadership (Monzani et al., 2021).

#### Authentic leadership

Authentic leadership is a positive leadership style that is grounded on other well-established positive leadership theories, such as transformational, ethical, and servant leadership (Avolio and Gardner, 2005), and that has become increasingly influential in recent years (Avolio et al., 2004; Walumbwa et al., 2008). Similarly, to how the gender equality movement started challenging the meaning of gender role stereotypes, the positive leadership movement challenged the attributes and behaviors that would describe an effective organizational leader (leader role stereotype; Monzani and Van Dick, 2020). More precisely, due to the destructive role that business leaders played after the 2008 wall street crash (Gandz et al., 2010; Crossan et al., 2017), the traditional managerial view on leadership, focusing on contingent rewards and punishments (“carrots and sticks”), lost ground to positive leader attributes and behaviors that advance the organizational goals by promoting their followers’ self-actualization and well-being. Authentic leadership style itself is described by two self-based psychological mechanisms; self-awareness and self-regulation (Gardner et al., 2005), and is operationalized through four dimensions: Firstly, s*elf-awareness* refers to the awareness of goals, emotions, and needs of both oneself and others. Secondly, *balanced processing of information* refers to the consideration of different stakeholders’ viewpoints before making important decisions. Thirdly, *relational transparency* refers to the establishment of open and clear relations with others. Lastly, *internalized moral perspective* refers to acting coherently with inner values, even in adverse contexts (Gardner et al., 2005). A myriad of studies has shown how authentic leadership predicts individual performance and loyalty above and beyond transactional leadership (Monzani et al., 2014, 2015a,b); negatively predicts employee silence (Monzani et al., 2016) and positively predicts managerial voice (Monzani et al., 2019). Further, authentic leadership predicts growth-enhancing social exchange between leaders and followers, which promotes their mutual well-being (Ilies et al., 2005; Weiss et al., 2018), and a variety of positive work outcomes, such as trust in leader and job satisfaction (see Gardner et al., 2011; Banks et al., 2016; Hoch et al., 2018).

In the realm of gender and leadership, it is important to note that authentic leadership can be seen as an androgynous leadership style (Monzani et al., 2015a,b, 2021). Authentic leadership captures agentic *and* communal leadership behaviors. Characteristics such as a higher awareness of followers’ developmental needs, developing growth-enhancing relations through open and transparent communication, and by considering others’ voice in decision-making are more congruent with the nurturing connotation of the female gender role (Monzani et al., 2021). Although authentic leaders can be caring and concerned for developing followers, authentic leadership also includes more agentic connotated behaviors, for instance when leaders are expected to act against strong situational or social pressures to defend their internalized values. In other words, doing “the right thing,” even if unpopular, demands a high level of assertiveness and dominance, which are agentic attributes.

There is some initial evidence suggesting that authentic leadership seems to be an alternative for female leaders to overcome the hurdles created by role incongruity. More precisely, women scoring higher in authentic leadership tend to identify more with their organizations and are also less likely to make unethical business decisions (Monzani et al., 2015a,b, 2021). These two constructs, in turn, are likely to reduce female leaders’ turnover intentions, and help them overcome the harsher scrutiny of their judgment calls by peers and followers that results from the double standard predicted by RCT.

Despite these encouraging findings, RCT suggests that, due to their minority status in male-dominated top management teams or executive boards, female managers may face more difficulties in achieving the relational authenticity required for being authentic leaders (Eagly, 2005). A recent study provided a complementary explanation, suggesting that the leader-gender role incongruence produces an attribution bias that affects female managers’ self-reports of authentic leadership behaviors (“*the authentic-female attribution bias*”; Monzani et al., 2015a,b).

“At the workplace, based on the female gender role, women are expected to show concern for others by (1) being highly aware of their needs and values (self-awareness), (2) to be relationship-oriented and developing open relations with others, and (3) to be emphatic and to consider different viewpoints (balanced processing of information), but as managers they are expected to be more agentic and act coherently with inner values (internalized moral perspective dimension of authentic leadership) even in adverse contexts. Because of this role conflict, female managers should attribute their self-awareness, balanced processing of information, and relational transparency to their gender role and not to their leadership role, perceiving themselves less authentic as leaders.” (Monzani et al., 2015a; Monzani et al., 2015b, p. 739). In other words, we propose that women do not behave less authentic as leaders than men, but because the gender role expectation for females is highly congruent with the communal aspects of authentic leadership, female managers themselves and also their followers tend to attribute these leadership behaviors to being a woman, while for male leaders, it would be attributed to being an authentic leader.

Following this line of thinking, we believe that (a) women themselves show this bias and ascribe less authentic leadership to themselves, and (b) followers also show this bias and ascribe less authentic leadership to female leaders. Based on this logic, we formulate the following hypotheses:

*Hypothesis 1a*: Women ascribe less authentic leadership behavior to themselves than men.

*Hypothesis 1b*: Female leaders are ascribed less authentic leadership behavior by their employees compared to male leaders.

### Group dynamics in leadership

Social psychologists defined leadership as “a process of social influence through which an individual enlists and mobilizes the aid of others in the attainment of a collective goal” (Chemers, 2001, p. 376). Therefore, leaders, by definition, cannot exist without followers, and often, leadership effectiveness is defined by the leaders’ influence on their employees. Thus, in reality, leaders act within social groups (e.g., their teams, departments, etc.), and “leaders not only lead groups of people, but they are also themselves members of these groups” (van Knippenberg & Hogg, 2003, p. 244). Therefore, leaders’ social identity, understood as “the individual’s knowledge that he/she belongs to certain social groups together with some emotional and valuable significance to him/her of this group membership” (Tajfel, 1972, p. 292), plays an important role for leaders’ acceptance and effectiveness (Ashforth and Mael, 1989; van Knippenberg and Hogg, 2003).

As mentioned above, a group’s prototype matters to determine “who will lead and who will follow (in this group).” When defining group boundaries and characteristics, social comparison and categorization are core processes (self-categorization theory; Turner et al., 1987). For these categorization and comparison processes, individuals use prototypes as mental heuristics (Hogg, 2001). Prototypes are defined as “fuzzy sets of attributes that define and prescribe attitudes, feelings, and behaviors that characterize one group and distinguish it from other groups” (Hogg, 2001, p. 187). The Social Identity Model of Leadership (SIMOL; Hogg, 2001; van Knippenberg et al., 2004) explains how these underlying social identity and social categorization processes act in leadership emergence and effectiveness.

The SIMOL focuses on how leaders operate within social groups and the characteristics of the leader *as a group member* (van Knippenberg and Hogg, 2003). One key factor for leaders’ effectiveness is their group prototypicality (van Knippenberg, 2011). When the group perceives the leader as representing the group’s prototype, they will see him or her as one of them (ingroup), interpret his or her behavior positively and assume he or she is acting in favor of the ingroup (van Knippenberg and Hogg, 2003). Therefore, in organizations, followers (e.g., employees) tend to like and trust a prototypical leader more than non-prototypical leaders, they are more likely to tolerate the shortcomings of a prototypical leader and ascribe them a higher degree of leadership effectiveness (van Knippenberg and Hogg, 2003; van Knippenberg and van Knippenberg, 2005; Giessner and van Knippenberg, 2008; van Dijke and De Cremer, 2008; Giessner et al., 2009; Ullrich et al., 2009; Steffens et al., 2021). Two recent meta-analyses have confirmed the positive effects of leader group prototypicality for a range of individual and organizational constructs (Barreto and Hogg, 2017; Steffens et al., 2021). Because prototypical leaders are seen as protecting and advancing the interests of the groups they lead, we expect that team prototypicality positively relates the positive leadership styles including authentic leadership and trust in leader.

Thus, in the present study, we propose that high team prototypicality, that is when the leader resembles or embodies the group’s prototype, will positively influence both the leaders’ self-perceptions and their perception by followers. Leaders who perceive themselves as prototypical for the team should feel enabled to show more authentic leadership and act authentic in their leader role. Leaders who are perceived acting in the interest of the group are generally perceived as more authentic and showing authentic leadership (Steffens et al., 2016). High team prototypicality should positively relate to more (pronounced) authentic leadership and more trust in leader. This logic leads us to formulating the following hypotheses:

*Hypothesis 2*: Leaders’ team prototypicality positively relates to authentic leadership behavior, both self-reported (H2a) and as rated by the followers (H2b).

*Hypothesis 3*: Leaders’ team prototypicality positively relates to employees’ trust in their leader.

### Gender and group dynamics in leadership

Now, why should prototypicality be a key factor for female leaders to overcome role incongruity issues? Societal barriers, such as gender and leader role stereotypes, that prevent women and other equity-seeking groups from climbing a firm’s hierarchical structure, are difficult to modify. Unfortunately, these stereotypes reflect the longstanding belief systems of a given society. In contrast, a group’s prototype is not stable *per se*, as it depends on how the group manages the categorization processes and comparison possibilities of its members, and, thus, is inherently more fluid (Turner et al., 1987; Monzani et al., 2015a,b). Therefore, team prototypicality is a fluid property of social groups, which can be modified more easily both by organizations and leaders themselves, for example, by engaging in identity entrepreneurship behaviors (Steffens et al., 2014; van Dick et al., 2018). Combining team prototypicality and leader gender, we assume that female leaders will benefit more from high team prototypicality than male leaders. Male leaders are not confronted with biases based on role incongruity as female leaders are and experience a better fit with the leader prototype. Team prototypicality shifts the comparative dimensions from assessing a match between leader gender and leader prototype to a match between the individual leader and the team members. Therefore, female leaders who perceive themselves as representing the group they lead or who are perceived by their followers as representing the group should be evaluated based on this congruity with the team which, in turn, should reduce biased perceptions and evaluations due to the role incongruity between the female gender role and the leader role.

Thus, we argue that if the salience of a female leaders’ team prototypical status is increased within the workgroup they lead, female leaders will not evaluate and not be evaluated by their followers based on societal stereotypes about gender and leader, but be perceived as representatives of the group they lead. In short, we claim that by leveraging the insights of SIMOL theory, we can expect to overcome the “double bind” that female leaders face. Therefore, we assume that the effects of high team prototypicality are stronger for female than for male leaders.

*Hypothesis 4: Team prototypicality and leader gender interact in a way that the relation between team prototypicality and authentic leadership is stronger for female leaders both in terms of their self-perception (H4a) and employees’ perception (H4b).*

*Hypothesis 5: Team prototypicality and leader gender interact in a way that the relation between team prototypicality and trust in leader is stronger for female leaders.*

### Follower outcomes

**Figure 2:**
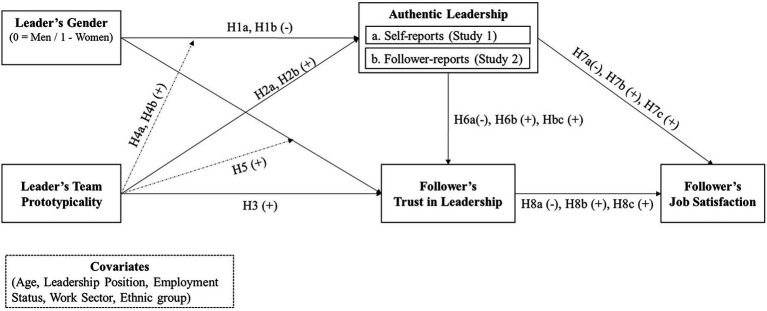
Extended theoretical model.

The right part of our extended theoretical model (see Figure 2) shows followers’ job satisfaction as the main individual outcome of our extended model. While the antecedents and mechanisms predicting job satisfaction have been studied thoroughly in the past, this construct remains a worthy metric for line and talent managers alike (Judge et al., 2020). A meta-analysis by Barreto and Hogg (2017) showed that group prototypicality is a significant predictor of trust in leader. In addition, there is extant evidence on the authentic leadership literature to predict direct and indirect effects on job satisfaction and trust in leader (Gardner et al., 2011;Banks et al., 2016; Hoch et al., 2018). Further, from previous research we know that trust in leader is related to job satisfaction (Gilstrap and Collins, 2012; Braun et al., 2013; Gibson and Petrosko, 2014).

Prior studies substantiated a main effect of prototypicality on authentic leadership. For example, leaders that are perceived as championing the interest of a collective are rated as being more authentic by their followers (Steffens et al., 2016). In turn, several studies show that authentic leadership is a strong correlate of both trust in leader and job satisfaction, with meta-analytic correlations ranging between *r* = 0.65 and *r* = 0.69 for trust in leader and ranging between *r* = 0.48 and *r* = 0.53 (Banks et al., 2016; Hoch et al., 2018). Finally, early studies and reviews in the field of authentic leadership have shown that trust in leader actually mediates followers’ perceptions of authentic leadership and job satisfaction (Wong and Cummings, 2009; Gardner et al., 2011; Černe et al., 2014). Building up on and combining these findings, we expect that team prototypicality has direct and indirect effects on job satisfaction as carried through authentic leadership and trust in leader, respectively.

We acknowledge that our hypotheses in this regard are rather of confirmatory, than of exploratory nature. However, we believe that replicating prior findings through hypotheses testing is important, as replication is a crucial part of the scientific process, and testing these hypotheses does not reduce the novelty of our study, which clearly lies (with) in our core model (see Figure 1) and the interactive effect of exogenous (biological sex) and endogenous (team prototypicality) antecedents of authentic leadership. Thus, combining these findings with our core model, we predict:

*Hypothesis 6*: Authentic leadership will mediate the indirect effect of (a) team prototypicality, (b) leader gender, and their (c) joint effect on trust in leader and job satisfaction.

*Hypothesis 7*: Trust in leader will mediate the indirect effect of (a) team prototypicality, (b) leader gender, and their (c) joint effect on job satisfaction.

*Hypothesis 8*: Authentic leadership and trust in leader will serially mediate the indirect effect of (a) leader gender, (b) team prototypicality, and their (c) joint effect on job satisfaction.

Adding these hypotheses to our core model (Figure 1) leads to the extended theoretical model (see Figure 2).

To test our hypotheses, we conducted two studies complementing each other. To test our core model (hypotheses H1 to H5), we first conducted an online experiment using a sample of US employees who also had leadership experience, in which we used a similar manipulation of team prototypicality as Monzani et al. (2015a,b) and asked them to report their authentic leadership behavior. Second, testing both our core and extended model, we conducted an online survey study with US employees who were asked to report their leader’s gender, perceived team prototypicality and authentic leadership, their trust in leader, and their job satisfaction.

## Study 1

### Study 1: Methods

#### Participants and procedure

Study 1 consisted of an online experiment using scenarios with a single-factor between-subject design (factor: low vs. high team prototypicality). We used the online survey program Unipark of Tivian and data were collected *via* Amazon Mechanical Turk (in the following referred to as MTurk). MTurk allows recruiting and compensating participants more efficiently than other data-collection approaches. Moreover, we chose MTurk as (a) it facilitates data acquisition of participants from the population of working adults who have or have had leading responsibilities in the present or past, and (b) as MTurk offers demographic diversity and good data (Buhrmester et al., 2011; Goodman et al., 2013). Participants should be currently working and have or have had a leading position in the past. Participants were informed that the survey would examine how leaders behave in different situations and would take about 10 min on average. They received 0.75 US$ as a compensation for their participation. The study was exempt from ethics approval at Goethe University Frankfurt as it passed all major ethical criteria for research (anonymity, voluntariness, etc.). As it is recommended for researchers using MTurk to screen for participants’ attention (Goodman et al., 2013), out of 430 individuals who opened/accessed the link to our study, we excluded all participants who did not provide a complete data set and/or those whose survey duration was 0. The final sample consisted of 315 (114 females, 201 males) individuals with a mean age of 33.69 years (*SD* = 11.21). Of these, 148 held a leading position (167 had no leading responsibility) and more than two-thirds of the participants (231 individuals) had a permanent contract. Two-thirds (206 individuals) worked in the private and 109 in the public sector. The majority, almost 80%, were European-American, 7.6% were African-American, 7.0% were Asian-American, 5.1% were Hispanic, and 0.3% were native Americans.

Participation in the study was voluntary and all participants provided their informed consent. At the beginning of the experiment, all participants indicated their biological sex and several other demographic variables (e.g., age, working sector, and ethnic group). After reporting their demographic information, participants were randomly assigned to one of the two experimental conditions. This manipulation of team prototypicality has already been used by Monzani et al. (2015a,b). In both experimental conditions, participants were asked to imagine to be upper managers in a multinational organization. Participants then received the results of a recent internal HR survey, which compared the fit between their scores and the scores of their team members on six key elements of their organization (vision, mission, organizational values, and culture, strategy, work processes, and career development opportunities). Thus, participants saw figures showing a high fit (65%–91%) between them and their team members on these six dimensions (high team prototypicality) or a low fit (12%–32%, low team prototypicality condition). On the following page, participants rated themselves on team prototypicality and authentic leadership. A short debriefing was provided on the screen after all scales had been completed.

#### Measures

##### Leader team prototypicality manipulation

As discussed above, we used the team prototypicality manipulation from Monzani et al. (2015a) to Monzani et al. (2015b). Depending on the experimental condition, participants read a scenario and saw figures with either high (ranging from 65% to 91%; high prototypicality condition) or low (ranging from 12% to 32%; low prototypicality condition) levels of fit to their team members.

**Figure 3:**
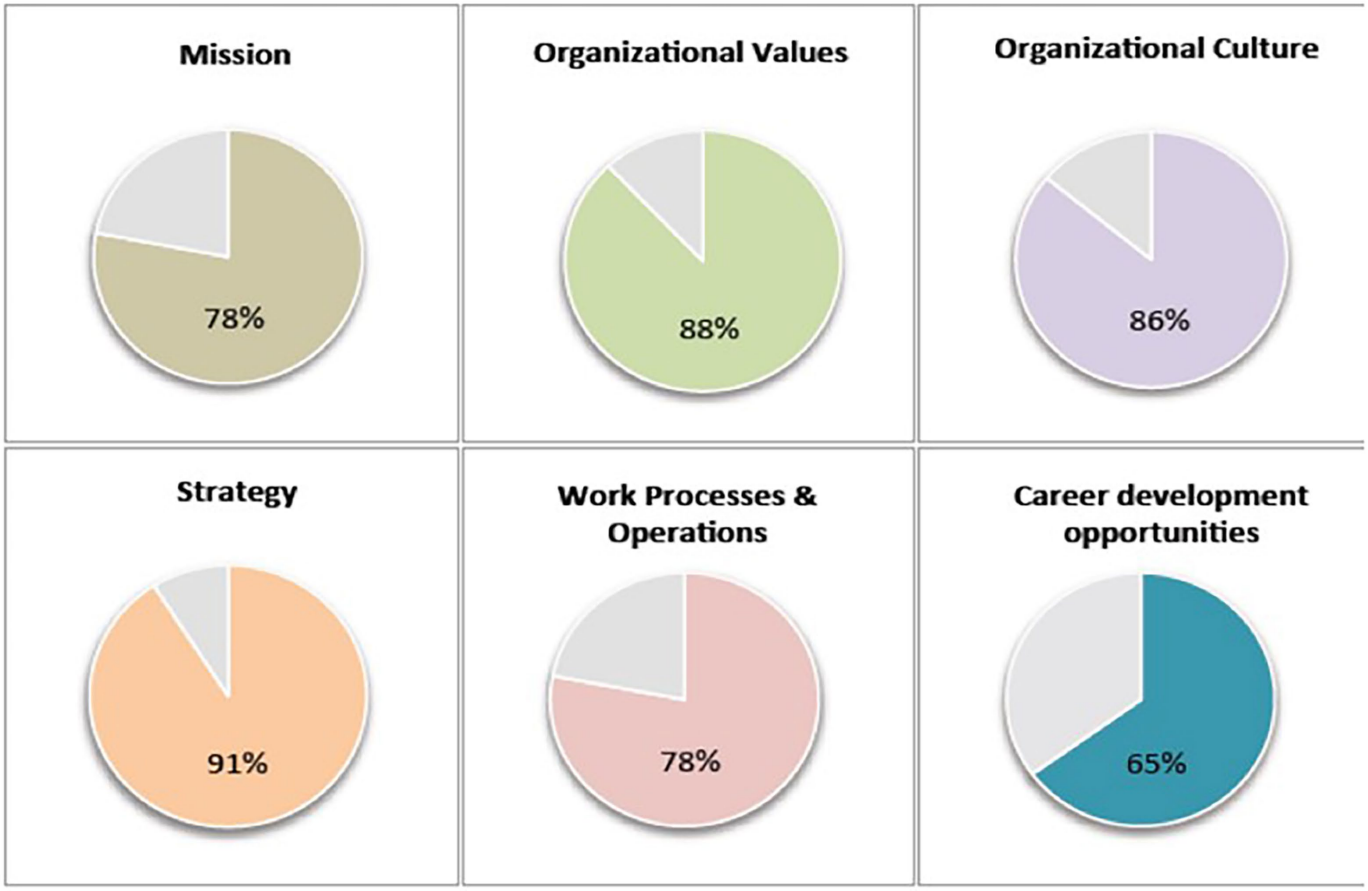
Team prototypicality manipulation (high team prototypicality condition) in Study 1.

Precisely, participants read the following:

“Now, imagine that you are high-level manager in the multinational organization EINROTH. You are leading a small team consisting of members from different areas of your organization, who are reporting directly to you. A few days ago, the results of an internal survey, performed by the Human Resource Department, were sent to your work e-mail address. The survey explored how both top and middle managers understand key elements of the organization, such as its mission, its values, how effective work processes are, or if professional development opportunities are present or not. The results of this survey matched the perceptions of the team leader (you) with the views of your team. For example, the match between your average scores and your team’s scores for which strategy this organization requires is 91% [24%]. Overall, these results show that you and your team have similar [different] views about the values and beliefs about this organization, its culture and how work should be done in order to be successful.” Below participants saw a figure depicting their match to their team members (see Figure 3).

##### Team prototypicality

Team prototypicality was measured with two items (adopted from Ullrich et al., 2009; Monzani et al., 2019) on a 7-point Likert scale with values ranging from *1 = do not agree at all* to *7 = fully agree*. The two items “I represent what is characteristic for my team” and “I represent what my team has in common.” showed an excellent reliability *(Cronbach’s* α = 0.95).

##### Authentic leadership

Authentic leadership was assessed with the Authentic Leadership Questionnaire (ALQ, Walumbwa et al., 2008). The ALQ consists of 16 items that assess the frequency of authentic behaviors in a leadership role, as captured by its four dimensions. Said behaviors were rated using a 5-point Likert scale with values ranging from *1 = not at all* to *5 = frequently*. Some exemplary items are “I say exactly what I mean” (Relational Transparency), “I make decisions based on my core values” (Internalized Moral Perspective), “I analyze relevant data before coming to a decision” (Balanced Processing of Information), and “I know when it is time to re-evaluate my position on important issues.” (Self-Awareness). The ALQ yielded excellent reliability (*Cronbach’s* α = 0.90).

##### Gender

Gender was assessed by asking participants for their biological sex (dummy coded, 0 = male, 1 = female).^1^

##### Controls

Several variables were assessed as potential control variables: age in years as proxy for work experience, employment type (0 = temporary, 1 = permanent), work sector (0 = public, 1 = private), leadership responsibility (0 = no, 1 = yes), and ethnic affiliation (0 = European-American, 1 = other).

A list of all variables and constructs assessed in Study 1 including the instruction, concrete items, and answer format can be found in Table A.1 in the Supplementary material.

#### Data analyses

Before testing our model, we conducted an ANCOVA with gender, age, kind of contract, sector, leadership responsibility, and ethnic affiliation (0 = European-American, 1 = other) as controls. The manipulation check revealed that the manipulation worked as intended and participants in the high team prototypicality condition (*M* = 5.604) perceived themselves as more prototypical for the team than those in the low team prototypical condition (*M* = 3.622, *F* (1,307) = 177.232, *p* < 0.001). In addition, we conducted a preliminary analysis to test our core model using an ANCOVA with age, kind of contract, sector, leadership responsibility and ethnic affiliation as controls, participant’s biological sex, and team prototypicality manipulation as between-factors. None of our factors nor their interaction term were significant (see Table 1).

**Table 1:** Study 1: Results of preliminary analyses with ANCOVA.

	Authentic leadership	
	*MS*	*F*
**Controls**		
Age	0.94	3.36^*^
Ethnic group^a^	0.05	0.18
Leadership position^b^	1.60	5.69^*^
Employment status^c^	1.19	4.24^*^
Work sector^d^	0.03	0.09
IVs		
Team prototypicality (manipulation)	0.01	0.04
Biol. sex^e^	0.20	0.69
Gender*team prototypicality	0.03	0.09
Model *R^2^*	0.06	

*N* = 315.

a 0 = European-American, 1 = other.

b Leading position, 0 = no, 1 = yes.

c 0 = temporary contract, 1 = permanent contract.

d 0 = public sector, 1 = private sector.

e Biological sex: 0 = male, 1 = female.

* *p* < 0.10;

* *p* < 0.05, two-tailed.

Thus, we employed a more sophisticated, two-stage data analysis strategy. First, we conducted multivariate regressions to explore our predictions regarding authentic leadership, and then replicated our analyses employing a more sophisticated, covariance-based approach, that is, structural equation modeling (SEM). We believe, this dual approach provides a good trade-off between the parsimony of our model and the robustness of our findings.

To test the hypotheses on self-perception (Hypothesis 1a, Hypothesis 2a, and Hypothesis 4a), we used model 14 of the PROCESS macro version 4.0 for SPSS with 10.000 bootstrapping samples, 95% confidence intervals. Team prototypicality as a metric variable of the interaction term was mean centered. Age, ethnic affiliation (European-American vs. other), possession of a leadership position (no vs. yes), employment status (limited vs. unlimited), and work sector (private vs. public) were entered as control variables. The team prototypicality manipulation was entered as independent variable, leader’s team prototypicality (scale) as mediator, biological sex as second stage moderator, and authentic leadership as dependent variable (see Table 2). In addition, we ran the model without any control variables, which did not change the pattern emerging, but slightly changed the magnitude of the results.

**Table 2:** Study 1: Results (manifest) with control variables (PROCESS model 14; 10.000 bootstrapping samples).

	Team prototypicality (scale)					Authentic leadership			
	*b*		*SE*	*t*		*b*		*SE*	*t*
Constant	−1.03	^**^	0.30	−3.48		3.64	^***^	0.12	30.23
Age	−0.01		0.01	−0.69		0.01	^*^	0.00	1.83
Ethnic group^a^	0.10		0.19	0.51		0.01		0.08	0.18
Leadership position^b^	0.23		0.15	1.55		0.13	^*^	0.06	2.18
Employment status^c^	0.40	^*^	0.17	2.36		0.13	^*^	0.07	1.84
Work sector^d^	−0.38	^*^	0.16	−2.38		0.04		0.06	0.68
Team prototypicality manipulation	1.98	^***^	0.15	13.30		−0.07		0.08	−0.92
Team prototypicality (scale)						−0.01		0.03	−0.25
Biological sex^e^						0.05		0.06	0.74
Biol. Sex*prototypicality (scale)					∆*R^2^* = 0.02^*^	0.09	^*^	0.04	2.53
	*R^2^* = 0.38^***^; *f ^2^* = 0.61; 1 − *β* = 0.999					*R^2^* = 0.08^***^; *f ^2^* = 0.09; 1 − *β* = 0.951			

*N* = 315. *f ^2 =^ Cohen’s f^2^; 1 − β =* Achieved statistical power.

a Leadership position, 0 = no, 1 = yes.

b 0 = temporary contract, 1 = permanent contract.

c 0 = public sector, 1 = private sector.

d 0 = European-American, 1 = other.

e 0 = male, 1 = female.

* *p* < 0.10;

* *p* < 0.05;

** *p* < 0.01;

*** *p* < 0.001, all two-tailed.

Therefore, in the second stage, we constructed structural equation models without controls in MPlus 8.2, following the recommendations by Kline (2013). Moreover, because simulation studies have shown that the chi-square (χ2) test is sensitive to sample size, we employed the mainstream additional goodness-of-fit indicators suggested by Cheung and Rensvold (2002). Finally, we used a robust estimator, the weighted least squares mean and variance adjuster (WLSMV) to prevent potential issues with non-normal distributions in structural equation modeling. These additional considerations allow us to ensure the robustness of our findings.

### Study 1: Results

Descriptive statistics, intercorrelations, and reliabilities are displayed in Table 3. Table 3 shows that, as predicted and confirmed by our manipulation check analysis, our experimental manipulation of team prototypicality was positively and strongly correlated with participants’ reports of (self-) perceived team prototypicality, suggesting that our manipulation evoked the intended effect. Further, also as expected, occupying a leadership position correlated positively with higher frequency of authentic leadership behaviors. Finally, the interaction term between participants’ biological sex and our prototypicality manipulation was related to authentic leadership, justifying our subsequent regression and SEM analyses.

**Table 3:** Study 1: Means, standard deviations, intercorrelations, and reliabilities.

	*M*	*SD*	(1)	(2)	(3)	(4)	(5)	(6)	(7)	(8)	
1. Age	33.690	11.21	–^f^								
2. Ethnic group^a^	0.20	0.40	−0.142^*^	–^f^							
3. Leading position^b^	0.47	0.50	0.095^*^	−0.001	–^f^						
4. Employment status^c^	0.73	0.44	0.109^*^	0.019	0.107^*^	–^f^					
5. Work sector^d^	0.65	0.48	0.097^*^	−0.047	0.096^*^	0.014	–^f^				
6. Leader’s biol. sex^e^	0.36	0.48	0.123^*^	−0.003	−0.007	0.081	−0.091	–^f^			
7. Leader team prototypicality manipulation	0.51	0.50	−0.073	−0.008	−0.002	−0.048	−0.008	−0.025	–^f^		
8. Perceived prototypicality (scale)	4.63	1.66	−0.071	0.030	0.067	0.081	−0.109^*^	0.047	0.595^***^	(0.95/^f^)	
9. Authentic leadership	3.98	0.54	0.135^*^	0.012	0.159^**^	0.147^**^	0.037	0.067	−0.031	0.059	(0.90/0.90)

*N* = 315. Internal consistency estimates (Cronbach’s α/McDonalds ω) are displayed on the diagonal in parentheses.

a 0 = European-American, 1 = other.

b Leading position, 0 = no, 1 = yes.

c 0 = temporary contract, 1 = permanent contract.

d 0 = public sector, 1 = private sector.

e Biological sex: 0 = male, 1 = female.

f Not applicable.

+ *p* < 0.10;

* *p* < 0.05;

** *p* < 0.01;

*** *p* < 0.001, all two-tailed.

**Figure 4:**
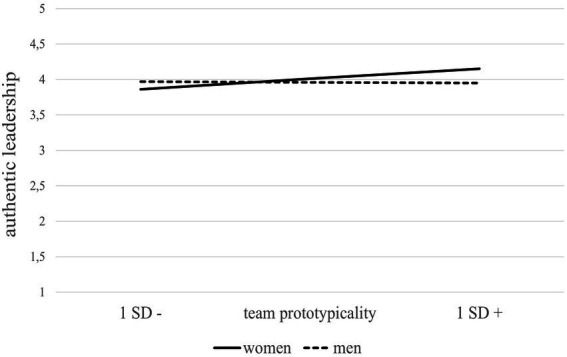
Study 1—Interactive effects between participants’ biological sex and team prototypicality manipulation (high vs. low) on authentic leadership.

The full model explained 8% of variance in authentic leadership behavior. The experimental condition significantly predicted team prototypicality (*b* = 1.98, *SE* = 0.15, *t* = 13.30, *p* < 0.001). Incongruent with hypotheses 1a and 2a, neither gender (*b* = 0.05, *SE* = 0.06, *t* = 0.74, *p* = 0.46) nor team prototypicality (*b* = −0.01, *SE* = 0.03, *t* = −0.25, *p* = 0.80) significantly predicted AL. However, and in line with Hypothesis 4a, the interaction term was significant (*b* = 0.09, *SE* = 0.04, *t* = 2.53, *p* = 0.01) and associated with a significant increase in explained variance (2%). For men, there was no significant conditional effect (*b* = −0.01, *SE* = 0.03, *t* = −0.25, *p* = 0.80), whereas for women it turned out significant (*b* = 0.09, *SE* = 0.03, *t* = 2.72, *p* = 0.007; see Figure 4). This applied for the mediation similarly: The mediation was significant for women (effect = 0.17, CI95%: 0.06, 0.29), but not for men (effect = −0.01, CI95%: −0.12, 0.09). Further, the index of the moderated mediation was significant (index = 0.19, CI95%: 0.06, 0.32).^2^ Figure 4 illustrates the interaction effect of team prototypicality and biological sex on authentic leadership.

#### Structural equation modeling

**Figure 5:**
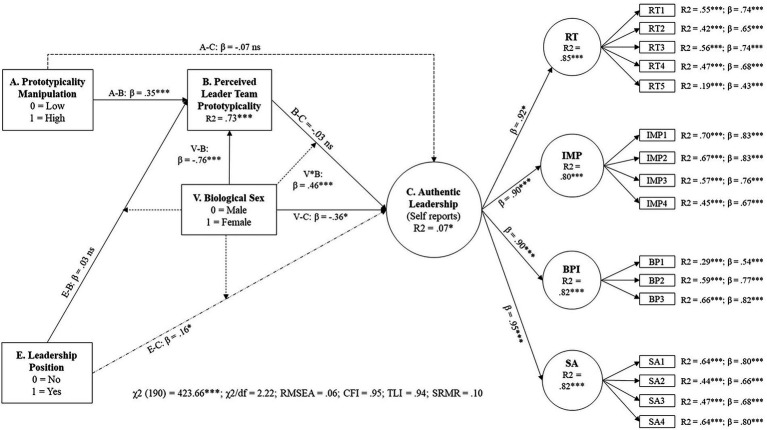
Study 1—Results of a SEM testing Hypotheses 1a, 2a, and H4a (self-perception perspective).

The results of our structural equation modeling align with our multivariate regression analyses. Both the measurement model [χ2(205) = 487.40, χ2/df = 2.38; RSMEA = 0.07, CFI = 0.95, TLI = 0.94, SRMR = 0.10] and the SEM [χ2(192) = 423.66, χ2/df = 2.22; RSMEA = 0.06, CFI = 0.95, TLI = 0.94, SRMR = 0.10] showed excellent fit to our data. The SEM model explained slightly less variance than our regression model (R^2^ = 0.07). As expected, the measurement model shows that our independent variables were uncorrelated, and all items of authentic leadership significantly loaded onto their respective dimensions with acceptable values. Only one item showed a loading lower than 0.50 (Item 5). In turn, each dimension showed significant loadings onto the higher-order construct (authentic leadership) with significant second-order loadings ranging from 0.89 to 0.95. The result pattern remained and further corroborated the results of the manifest analyses. Figure 5 illustrates the retained SEM model.

#### Additional manipulation checks

Our SEM model allowed us to conduct a more robust check regarding our experimental manipulation of (leader) team prototypicality. More precisely, occupying a leadership position might have elicited perceptions of team prototypicality in our participants. If that was the case, we would expect a main effect of leadership position on team prototypicality perceptions, or even an interactive effect with participants’ biological sex. In contrast, we did not detect any main or interactive effects of leadership position on perceptions of team prototypicality. Further, we tested if biological sex would moderate the effect of occupying a leadership position on perceptions of authentic leadership. Interestingly, after controlling for manipulated and perceived team prototypicality, this effect turned out non-significant. Although at first sight, this finding might seem trivial, it further demonstrates that the double bind and prejudice that women face in leadership roles is not a mere structural or quotas issue (occupying a position or not, regardless of gender), but also an issue of psycho-social nature (women, regardless of their leadership position, tend to report both lower team prototypicality and authentic leadership).

### Study 1: Discussion

Neither the preliminary analysis nor the more sophisticated analyses in Study 1 supported hypotheses 1a and 2a. There were gender differences in self-reported authentic leadership, nor did leaders’ team prototypicality influence AL. Although not visible in the preliminary analysis (very likely due to restricted variance of the dichotomous nature of the experimental condition team prototypicality factor), Study 1 provided initial support for our theorizing, in that high team prototypicality helps women feel more authentic when occupying a leader role. More precisely, when women perceived themselves as being prototypical for their teams—as induced by the experimental condition and confirmed by the manipulation check—they reported significantly higher AL. Additionally, our confidence in our findings is strengthened by the fact that we found this result/pattern both in manifest and latent analyses testing our hypotheses.

This study has some strengths and some obvious limitations. In terms of strengths, we employed exogenous variables as predictors, and randomly assigned participants to our experimental conditions. Due to these strengths in design, we do not anticipate concerns about potential endogeneity issues when making our causal claims (Antonakis et al., 2010). Similarly, because we used a manipulation, biological gender, and self-reports, at first glance, we do not have any concerns regarding common method variance (Podsakoff et al., 2012). Taken as a whole, these precautions increase our confidence in the robustness of our results. However, one limitation of Study 1 is that we only assessed and tested the effect of team prototypicality on self-perceived and self-reported authentic leadership, using a scenario-based online experiment. Therefore, participants’ answers might be influenced by ego-protective biases. Yet, self-perception only tells half of the story. Thus, to strengthen our argument and complement the findings of Study 1, we conducted an online survey, in which we assessed followers’ perceptions of their leader as well as their own attitudes.

## Study 2

In Study 1, we showed that women’s self-ratings in AL were positively affected by team prototypicality. Being assigned to the high team prototypicality condition lead to higher authentic leadership especially in female participants. Extending these results, in Study 2, we examined the effects of leaders’ team prototypicality and leader gender on other-reported authentic leadership behavior and employees’ trust in leader (Podsakoff et al., 1990; Burke et al., 2007).

### Study 2: Methods

#### Participants and procedure

Participants were US employees who participated in an online questionnaire that was programmed in the software Unipark by Trivian and the survey was then posted on Amazon’s Mechanical Turk platform addressed at currently working people. Despite its critics, some studies show that the practical benefits of these platforms (higher sampling heterogeneity and diversity, real working population vs. students), outweigh its limitations (Buhrmester et al., 2011). A total of 346 individuals participated in the survey. We deleted participants with missing data, the test trial participations from the research team members, and those with suspicious answer patterns (like always the same number—even in reversed items). Thus, our final sample consisted of 300 participants (111 female) with a mean age of 31.71 years (*SD* = 9.53). Eighty-eight participants had a leadership position themselves and 212 had no leading position. The majority (222 individuals) had a permanent contract and only 78 participants had a temporary contract. Almost 60% (176 participants) worked in the private sector, and 124 participants worked in the public sector. The majority (76.3%) were European-American, 10.3% were African-American, 9.0% were Asian-American, 4.0% were Hispanic, and 0.4% were native Americans.

At the beginning of the survey, participants indicated some demographical variables like participants’ biological sex, age, and their leaders’ biological sex. Then, they rated their leader’s authentic leadership behavior and team prototypicality. Afterward, employees rated themselves on some employee variables like trust in leader. Again, participation in the study was voluntary and all participants provided their informed consent. Participants received a US-$1 show-up fee for taking part of our study.

#### Study 2: Measures

##### Authentic leadership

As in Study 1, authentic leadership was assessed with the 16 items of the Authentic Leadership Questionnaire (ALQ, Walumbwa et al., 2008) on a 5-point Likert scale with values ranging from *1 = not at all* to *5 = frequently*. Because employees should rate their leader, we used the other-reports version instead of self-report version. Again, the ALQ had excellent reliability in this second dataset (*Cronbach’s* α = 0.94).

##### Team prototypicality

Four items (adopted from Ullrich et al., 2009) assessed the leaders’ team prototypicality rating. The four items were “He or she represents what is characteristic for my team.,” “He or she is a good example of the kind of people that are in my team.,” “He or she stands for what people who work in my team have in common.,” and “He or she is very similar to most people in my team..” Participants rated on a 5-point Likert scale with values ranging from *1 = strongly disagree* to *5 = fully agree* how much the items applied to their leader. The scale showed an excellent reliability *(Cronbach’s* α = 0.93).

##### Job satisfaction

Based on Spector (1985), we used five items to assess job satisfaction. Thereby, we asked participants how satisfied they are with (a) their salary, (b) their leader, (c) their colleagues, (d) the work itself, and I overall. Participants rated on a 5-point Likert scale their degree of satisfaction (*1 = very dissatisfied* to *5 = very satisfied)*. The mean of these items was used as a measure of overall job satisfaction. The scale showed good reliability (*Cronbach’s* α = 0.80).

##### Trust in leader

Trust in leader was assessed with three items (Podsakoff et al., 1990). The items were “I feel quite confident that my supervisor/leader will always treat me fairly,” “My supervisor/leader would never try to gain advantage by deceiving workers,” and “I have complete faith in the integrity of my supervisor/leader.” Participants indicated how much these items applied to their leader (*1 = strongly disagree* to *5 = fully agree*). The items showed excellent reliability (*Cronbach’s* α = 0.93).

##### Leader’s gender

Leader’s gender was assessed by asking participants for the biological sex of their leader (dummy coded, 0 = “male, 1 = “female”).

##### Controls

Participant’s gender was assessed by asking participants for their anatomical sex (dummy coded, 0 = male, 1 = female). Further, age in years as a proxy for work experience was included as control. Additional dummy coded controls were employment status (0 = temporary, 1 = permanent), work sector (0 = public, 1 = private), leadership responsibility (0 = no, 1 = yes), and ethnic affiliation (0 = European-American, 1 = other).

A list of all variables and constructs assessed in Study 2 including the instruction, concrete items and answer format can be found in Table A.2 in the Supplementary material.

### Study 2: Results

Descriptive statistics, intercorrelations and reliabilities are displayed in Table 4. For testing our core hypotheses H1 to H5, we used model 1 of PROCESS version 4.0 with 10,000 bootstrapping samples. Team prototypicality as metric variable of the interaction term was mean centered. Age, ethnic affiliation (European-American vs. other), possession of a leadership position (no vs. yes), employment status (limited vs. unlimited), and work sector (private vs. public) were entered as controls in all analyses.^3^ Authentic leadership was entered as dependent variable for testing hypotheses H1b, H2b, and H4b. Trust in leader was entered as dependent variable for testing hypotheses H3 and H5.

**Table 4:** Study 2: Means, standard deviations, intercorrelations, and reliabilities.

	*M*	*SD*	(1)	(2)	(3)	(4)	(5)	(6)	(7)	(8)	(9)	(10)	(11)
1. Age	31.71	9.53	–^f^										
2. Ethnic group^a^	0.24	0.43	−0.150^**^	–^f^									
3. Leadership position^b^	0.29	0.46	0.094	−0.083	–^f^								
4. Employment status^c^	0.74	0.44	0.098^*^	−0.117^*^	0.098^*^	–^f^							
5. Work sector^d^	0.59	0.49	0.116^*^	0.085	−0.009	−0.004	–^f^						
6. Participant’s biol. Sex^e^	0.37	0.48	0.170^**^	−0.053	−0.008	0.029	−0.142^*^	–^f^					
7. Leader’s biol. Sex^e^	0.40	0.49	0.110^*^	0.010	0.012	−0.059	−0.006	0.361^***^	–^f^				
8. Team prototypicality	3.81	0.94	0.093	0.010	−0.032	0.079	−0.017	0.051	−0.001	(0.93)			
9. Authentic leadership	3.69	0.76	0.033	0.028	−0.024	0.094	−0.064	0.109^*^	−0.039	0.737^***^	(0.94)		
10. Job satisfaction	3.59	0.79	0.136^*^	−0.077	0.084	0.091	−0.088	0.094	−0.081	0.541^***^	0.573^***^	(0.80)	
11. Trust in leader	3.84	1.10	−0.031	0.028	0.044^**^	0.116^*^	−0.074	0.089	−0.075	0.694^***^	0.781^***^	0.620^***^	(0.93)

*N* = 300. Internal consistency estimates (Cronbach’s α) are displayed on the diagonal in parentheses.

a 0 = European-American, 1 = other.

b 0 = no, 1 = yes.

c 0 = temporary contract, 1 = permanent contract.

d 0 = public sector, 1 = private sector.

e 0 = male, 1 = female.

f Not applicable.

* *p* < 0.10;

* *p* < 0.05;

** *p* < 0.01;

*** *p* < 0.001, all two-tailed.

#### Manifest testing of core model

##### Authentic leadership

**Figure 6:**
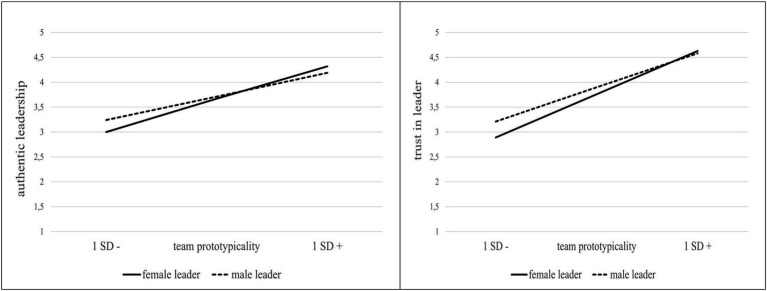
Study 2—Interaction between leader gender*team prototypicality predicting authentic leadership (left) and trust in leader (right).

The full model explained 56% of variance in authentic leadership (AL). Leader’s gender (*b* = −0.05, *SE* = 0.06, *t* = −0.81, *p* = 0.42) was not significant and hypothesis 1b was not supported. However, team prototypicality (*b* = 0.51, *SE* = 0.04, *t* = 12.07, *p* < 0.000) and its interaction with leader gender (*b* = 0.19, *SE* = 0.06, *t* = 2.98, *p* = 0.003) was significant. The interaction term was associated with a significant increase in explained variance (1.3%, *p* = 0.003, see Table 5). Subsequently conducted simple slope analyses showed that both slopes were significant; however, the slope for women was slightly steeper (*b* = 0.70, *SE* = 0.05, *t* = 14.81, *p* < 0.001) than the slope for men (*b* = 0.50, *SE* = 0.04, *t* = 12.07, *p* < 0.001; see Figure 6). Thus, hypothesis 2b was supported.

**Table 5:** Study 2: Results of hierarchical regression predicting authentic leadership and trust in leader (core model).

		Authentic leadership					Trust in leader			
		*b*		*SE*	*t*		*B*		*SE*	*t*
Constant		3.87	^***^	0.14	27.12		4.26	^***^	0.22	19.57
Participants’ age		−0.00		0.00	−0.96		−0.01	^*^	0.01	−2.37
Ethnic group^a^		0.04		0.07	0.60		0.06		0.11	0.56
Leadership position^b^		0.01		0.07	0.10		0.18	^*^	0.10	1.78
Employment status^c^		0.07		0.07	1.01		0.15		0.10	1.48
Work sector^d^		−0.07		0.06	−1.23		−0.11		0.09	−1.23
Team prototypicality		0.51	^***^	0.04	12.07		0.73	^***^	0.06	11.26
Leader’s biol. Sex^e^		−0.05		0.06	−0.81		−0.14		0.09	−1.47
Leader’s biol. Sex*team prototypicality	∆*R^2^* = 0.01^**^	0.19	^**^	0.06	2.98	∆*R^2^* = 0.01^*^	0.20	^*^	0.10	2.07
		*R^2^* = 56^***^; *f ^2^* = 1.27; 1 − *β* = 0.999					*R^2^* = 51^***^; *f ^2^* = 1.04; 1 − *β* = 0.999			

*N* = 300. *f^2 =^ Cohen’s f^2^; 1 − β =* Achieved statistical power.

a 0 = European-American, 1 = other.

b 0 = no, 1 = yes.

c 0 = temporary contract, 1 = permanent contract.

d 0 = public sector, 1 = private sector.

e 0 = male, 1 = female.

* *p* < 0.10;

* *p* < 0.05;

** *p* < 0.01;

*** *p* < 0.001, all two-tailed.

##### Trust in leader

The full model explained 51% of variance of trust in leader. Supporting hypothesis 3, team prototypicality (*b* = 0.73, *SE* = 0.06, *t* = 11.26, *p* < 0.000) was a significant predictor of trust in leader. Leader gender (*b* = −0.14, *SE* = 0.09, *t* = −1.47, *p* = 0.14) was not significant, but the interaction of team prototypicality and leader gender (*b* = 0.20, *SE* = 0.10, *t* = 2.07, *p* = 0.040) was significant. The interaction term was associated with a significant increase in explained variance (0.7%, *p* = 0.040, see Table 5). Subsequently, the simple slope analysis conducted showed that both slopes were significant, however, the slope for female leaders was slightly steeper (*b* = 0.93, *SE* = 0.07, *t* = 12.87, *p* < 0.001) than the slope for male leaders (*b* = 0.73, *SE* = 0.06, *t* = 11.26, *p* < 0.001; see Figure 6). Thus, hypothesis 5 was supported.

#### Testing the extended model with structural equation modeling

**Figure 7:**
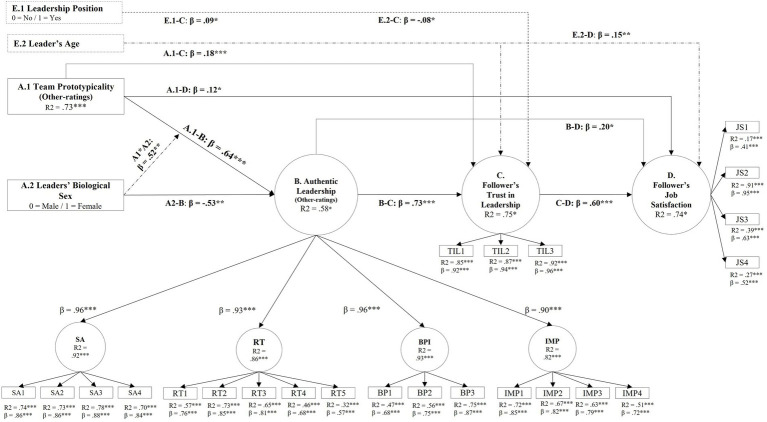
Study 2—SEM model illustrating direct and indirect effects of leader’s biological sex and team prototypicality on follower’s trust in leader and job satisfaction.

Both our measurement model [(χ2 (323) = 683.30, χ2/df = 2.12; RSMEA = 0.06, CFI = 0.97, TLI = 0.97, SRMR = 0.05)] and SEM models [(χ2 (205) = 487.40, χ2/df = 2.38; RSMEA = 0.07, CFI = 0.95, TLI = 0.94, SRMR = 0.10)] showed a good fit to our data. Figure 7 shows the standardized loadings and regression coefficients. Overall, the SEM model shows a similar pattern of results as those detected in our multivariate regression (except for hypothesis 1b). Further, we used the “INDIRECT” command in Mplus for obtaining standardized indirect effects in order to test our hypotheses H6a–H6c, H7a–H7c, and H8a–H8c.

Authentic leadership mediated the indirect effect of leader’s gender on trust in leader [β = −0.38 (0.11), *p* < 0.001]. Similarly, authentic leadership mediated the specific indirect effect of team prototypicality on trust in leader [β = 0.46 (0.04), *p* < 0.0001] and their joint effect [β = 0.38 (0.12), *p* < 0.002]. Similarly, authentic leadership mediated the specific indirect effect of leader gender on job satisfaction [β = −0.11 (0.05), *p* < 0.05] and the indirect specific effect of team prototypicality on job satisfaction [β = 0.13 (0.06), *p* < 0.03]. Similarly, the indirect specific joint effect of leader gender and team prototypicality as mediated by authentic leadership was also significant [β = 0.11 (0.06), *p* < 0.05]. These results provide support to hypotheses H6a, H6b, and partially support H6c.

In contrast, trust in leadership did not mediate the effect of leader gender on job satisfaction [β = 0.01 (0.10), *p* < 0.95]. Similarly, trust in leader did not mediate the joint effect of leader’s gender and team prototypicality on job satisfaction [β = −0.02 (0.11), *p* < 0.86]. Yet, trust in leadership mediated the specific effect of team prototypicality on job satisfaction [β = 0.11 (0.03), *p* < 0.001]. These results only provide support for hypotheses H7b, but not H7a nor H7c.

Finally, we explored the serial mediation effects of authentic leadership (first stage mediator) and trust in leader (second stage mediator) on job satisfaction. Again, both authentic leadership and trust in leadership significantly mediated the indirect effect of leader gender [β = −0.23 (0.08), *p* < 0.003] and of team prototypicality [β = 0.28 (0.04), *p* < 0.0001]. Finally, these two constructs mediated the joint effect of leader gender and team prototypicality on job satisfaction [β = 0.23 (0.08), *p* < 0.006]. Taken together, these results provide support for hypotheses H8a, H8b, and H8c.

### Study 2: Discussion

The SEM analyses supported Hypothesis 1b as there were substantive gender differences in authentic leadership in the extended model. However, H1b was not supported in testing the core model H1b. Hypothesis 2b was supported in testing of both the core model and the extended model, as team prototypicality was positively related to follower-reported authentic leadership. In addition, this relation was moderated by leader gender and was stronger for female leaders than for male leaders, as found in both the testing of the core and the extended model. Thus, hypothesis 4b was supported. Leaders’ team prototypicality helped female leaders to be perceived as authentic leaders by their employees. Further, the regression analyses testing our core model showed that team prototypicality positively related to trust in leader (supporting Hypothesis 3) and this effect was moderated by leader gender and, as expected, was stronger for female leaders (supporting hypothesis 5).

In addition, the results of our SEM model show that the interactive effect of leader gender and perceived team prototypicality on trust in leader, was mediated by authentic leadership^4^. Thus, our mediation hypotheses for authentic leadership were supported (H6a, H6b) or partially supported (H6c). This result aligns with the findings of Steffens et al. (2016), which suggest that for leaders to be deemed authentic, at first, they need to embody the prototype of the group they lead, but also be seen as advancing and protecting its interests.

Our study also shows that team prototypicality increases the authenticity of female leaders in their followers’ eyes, which, in turn, increases their trust and, eventually, their job satisfaction. This fact is evidenced in the single slopes of our multivariate regression and the positive sign of the joint indirect effect on trust in leader and job satisfaction (supporting hypotheses 8a, 8b, and 8c).

The fact that trust in leader alone did not mediate the effect of leader gender on job satisfaction is an intriguing finding of our study. It evidences the double-bind and prejudice that female leaders face, as suggested by RCT. By isolating specific indirect effects on a SEM mode, we can evince how female leaders are trusted less by their followers and how the latter report lower levels of job satisfaction, as well. Luckily, our second study also illustrates the value of both team prototypicality and authentic leadership for transcending these unfortunate biases.

## General discussion

The main goal of the present study was to explore if team prototypicality (i.e., being representative of the team one is leading) can reduce prejudice and double standards that women face when occupying a leadership role. We grounded our predictions in Role Congruity Theory (Eagly and Karau, 2002) and the Social Identity Model of Leadership (Hogg, 2001). Based on prior findings, our core claim was that high team prototypicality—although generally associated with positive effects for leaders—might be a key for women and female leaders to overcome role incongruity issues. We argue that team prototypicality shifts the evaluation frame from higher-order categories like gender and leader roles away to more concrete, group-related aspects and thereby reduces biases that stem from the incongruity between the female gender role and the leader role. This process should affect both self-perceptions and perceptions by followers. Female leaders who perceive themselves as representative of the team and those who are perceived as representative of the team by their followers should score higher on authentic leadership and be trusted more. Indeed, we found support for this rationale in both studies.

We chose authentic leadership due to its combination of communal and agentic connotated behaviors to examine the potential of team prototypicality for female leaders. Despite its androgynous character, we assumed to find the general think manager-think male pattern reflected in higher scores in authentic leadership for male compared to female leaders (Hypothesis 1). However, regarding Hypothesis 1, we found mixed results. H1 was not supported in self-perception but could be supported for follower-perception in the SEM testing of the extended model. A potential reason might lie in the androgynous nature of authentic leadership and the ongoing development of leadership roles becoming less masculine, as evidenced in more recent studies, i.e., by Koenig et al. (2011). This might reduce gender and leader role driven differences between female and male leaders.

In line with the SIMOL and previous research (Giessner and van Knippenberg, 2008; Giessner et al., 2009; Ullrich et al., 2009; van Knippenberg, 2011), in Study 2, team prototypicality positively related to authentic leadership (H2b) and trust in leader (H3). Yet there was no relation between prototypicality and the self-ascription of authentic leadership in Study 1 (H2a). One possible explanation could be that the scenario induced team prototypicality (low vs. high) evokes a weaker impact than actually experienced team prototypicality in reality.

Our results supported Hypotheses 4 and 5, which predicted that the positive relations of team prototypicality and authentic leadership as well as trust in leader are stronger for female than for male leaders. The relation between team prototypicality and authentic leadership was stronger for women (H4) and they benefitted more from high team prototypicality. This pattern was stable among both self-perceptions (Study 1) and follower ratings (Study 2). Further, a similar pattern was found for the relation between team prototypicality and trust in leader (H5; Study 2). Female leaders profit more, compared to male leaders, when their followers perceive them as highly prototypical for the team.

Finally, our SEM model provided full or partial support for the indirect joint effect of leader gender and team prototypicality in two important follower outcomes, trust in leader and job satisfaction. This is not a trivial finding, as indirect effects capture the otherwise hidden synergies among constructs. In our second study, we found moderate to large indirect effect sizes, which is not common in the literature when testing (serial) mediation models.

In summary, these results highlight the relevance of considering individual and contextual factors in female leadership research (i.e., being authentic and prototypical). For example, Gloor et al. (2020) showed that teams’ gender diversity influences the evaluation of female and male leaders in a way that if there is a higher proportion of women, female leaders are perceived as more prototypical. Our research even goes beyond this pure gender-based definition and perception of general prototypicality and shows that team prototypicality—which is more proximal and amenable by both the leader and the organization—seems to be one crucial factor for women overcoming obstacles based on role incongruity and a promising venue for future research and interventions.

### Theoretical implications

Our work provides a theoretical contribution to gender inequality by combining RCT and SIMOL theories. We provide initial evidence on how the in-group phenomenon, such as team prototypicality, can reduce the negative effects of traditional, societal gender role stereotypes on the advancement of women into leadership positions. In line with the point of Eagly (2005), if we are to reduce the prejudice and discrimination that women suffer, we need to find new ways to challenge a group’s prototype of what an “effective leader” looks like, so that it is no longer based on traditional role stereotypes. Thus, focusing on team prototypicality and the (perceived) match of the leader to its followers—not based on gender, but on more dimensions—reduces the biases based on gender and leader role incongruity. Whereas organizational or industry-related prototypicality per definition are focusing on being representative for more distal, higher-order categories, team prototypicality shifts the focus to more proximal, team-, value-and work-related categories. We believe that this shift also leads to a change in the evaluation frame that both individuals use to evaluate themselves as team leaders and that is used by others to evaluate their team leaders. Thereby, mismatched perceptions of female leaders that stem from the incongruity between higher-order categories of the male connotated leader and the female gender role stereotype should be reduced. Further, we believe that such a shift toward the group and team as evaluative frame might be a chance not only for female leaders, but also for minority members who face discrimination and biases due to the use of higher-order social categories like ethnic background. Therefore, we encourage future research to examine the potential of team prototypicality to reduce social category-based biases in the realm of leadership beyond gender.

In this paper, we combined two conceptions of leadership: a leader-oriented approach (authentic leadership) and a group-oriented approach (SIMOL). We chose authentic leadership due to its androgynous conception and inclusion of both communal and agentic behaviors and its well-established positive outcomes (Gardner et al., 2011; Banks et al., 2016; Hoch et al., 2018). We chose the SIMOL due to its focus on group dynamics and its positive outcomes (Barreto and Hogg, 2017; Steffens et al., 2021). As outlined in the previous paragraph, we believe that when focusing on the team level, team prototypicality might shift the comparative dimension that is used for the evaluation of leaders and, thereby, allows to reduce biases based on the incongruity between the leader and female gender role. As assumed and shown, neither approach on its own is sufficient to break the double bind that women suffer in leadership positions. However, following the theoretical rationale provided by Eagly (2005), the present study shows that when these two complementary perspectives are considered together, their joint effect contributes to overcoming the barriers that role stereotypes create for female leaders. So, if women perceive themselves but also are perceived as prototypical of the team they are leading, they will likely show a higher frequency of authentic leadership and are trusted more by their followers. Thus, the general positive consequences of team prototypicality are reinforced for female prototypical leaders as they benefit from the shift of the comparative frame more than men do. In this regard, we heed Avolio’s (2007) call to promote more integrative strategies in the leadership research and practice.

From the above, it follows that embracing a more holistic view of leadership is a promising avenue for future research, especially when examining gender equality in leadership. The Social Identity Model of Leadership has been recently expanded into the Identity Leadership framework by Haslam et al. (2021). The identity leadership framework suggests that in addition to prototypicality, leaders can be effective by shaping their group’s sense of a common identity by three paths, namely advancement, entrepreneurship, and impresarioship. Steffens et al. (2014) developed the identity leadership inventory to measure these aspects of identity leadership and recently van Dick et al. (2018, 2021) validated the inventory in a global study across 30 different countries on all continents. As the other dimensions of the ILI also focus on the leader as team member and acting in the group’s interest, future research should examine if other dimensions of identity leadership can also be converted into an advantage for female leaders—either alone or in combination with prototypicality. Further studies might attempt to replicate our findings in larger cross-cultural samples, to avoid the pitfall of generalizing insights of Western cultures into other cultures that might differ in their values, and thus also differ in their traditional role stereotypes (Obioma et al., 2021).

### Practical implications

Our research shows the potential of team prototypicality as a facilitator for female leaders. One advantage of team prototypicality is that it is not a stable construct, but it is malleable and influenced by group dynamics. Thus, team prototypicality perceptions can be increased by leaders themselves by actively being entrepreneurs of their identity (Reicher et al., 2005), e.g., *via* increasing their perceived team prototypicality by approval-seeking out-group behaviors or by reconstructing the social context by creating intergroup competition (van Knippenberg and Hogg, 2003). Although we think that this approach can be a promising one for female leaders to reduce biases based on role incongruity, in our view the burden should not lie in the hand of female leaders. Moreover, organizations and upper management could support the team prototypicality perceptions of female leaders by subtle highlighting the fit of the (female) leaders and the work group they are leading.

Further, if an in-group phenomenon, such as team prototypicality, can reduce the effects of traditional societal role stereotypes, then something similar might occur within an organization’s culture. Consequently, organizations can take action to shape a positive and inclusive culture, even in societies with strong traditional role stereotypes. When organizational culture acknowledges and values both masculine and feminine connoted attributes in their leaders, such a positive organizational culture would reduce general biases based on role congruity for female leaders—in both their self-perception and the perception by their employees and coworkers—independent of the team prototypicality.

Finally, our findings also have implications for project managers and team leaders who operate in firms whose cultures promote toxic masculinity (e.g., investment banking, military, etc.). Again, if in-group dynamics can over-ride the negative effect of a toxic organizational culture in their employees, then project managers and team leaders have the possibility to take action by (re)shaping the prototype of the workgroup and values of the workgroup so that it becomes more inclusive with regards to women (and other minorities) occupying a leadership role.

### Strengths and limitations

Our research is a first step, but of course, future research could build on these results and overcome some limitations. First, future studies should examine the effect of different manipulations of team prototypicality, as in the present study manipulation was solely based on work-related characteristics. Future research should examine the effects of team prototypicality by using manipulations that include personal values, personality, or a combination of different aspects. Second, we exclusively focused on prototypicality at the team level. Although we believe that team prototypicality is the most promising focus of prototypicality to override or reduce biases and discrimination based on the more abstract categories of gender and leader roles, future research should also examine the properties of other foci of prototypicality (e.g., organizational prototypicality). Third, we used cross-sectional survey data in Study 2. This design *per se* does not allow to draw causal conclusions and might be prone to common method bias. Yet, as we find similar patterns in both the experimental (Study 1) and the cross-sectional (Study 2) design, used different indicators (e.g., manipulation, self-reports) and the biological sex of the leader as key variables in both studies, which should not be influenced by common method bias (Podsakoff et al., 2003, 2012), in our opinion, the risk of false conclusions based common-method bias is very small. However, future research should replicate our findings by using multi-level data to compare the effects of leaders’ self-perceived prototypicality and leaders’ prototypicality perceived by the employees of female compared to male leaders. Further, using longitudinal data would allow tracking the evolvement of team prototypicality perceptions – contingent of certain leader behaviors – and its effects over time. Despite the limitations, the fact that we replicated our findings across two studies involving different perspectives, makes us confident in our results.

## Conclusion

Statistics show that even after 20 years of academic insight on how to reduce gender inequality in leadership, there is still a long way to go. The present study provides evidence on how ingroup dynamics in form of team prototypicality can support leaders, especially female leaders, to unleash their true leadership potential.

## Data availability statement

The raw data supporting the conclusions of this article will be made available by the authors, without undue reservation.

## Ethics statement

Ethical review and approval was not required for the study on human participants in accordance with the local legislation and institutional requirements. The patients/participants provided their written informed consent to participate in this study.

## Author contributions

AH, LM, and RD contributed to the development and design of the research. AH organized data collection, performed the initial statistical analyses and all manifest analyses, wrote the first draft of the manuscript and its revisions. LM performed the latent analyses. Both LM and RD revised the first draft of the manuscript critically and checked the revised versions. All authors contributed to the article and approved the submitted version.

## Conflict of interest

The authors declare that the research was conducted in the absence of any commercial or financial relationships that could be construed as a potential conflict of interest.

## Publisher’s note

All claims expressed in this article are solely those of the authors and do not necessarily represent those of their affiliated organizations, or those of the publisher, the editors and the reviewers. Any product that may be evaluated in this article, or claim that may be made by its manufacturer, is not guaranteed or endorsed by the publisher.

## Footnotes

^1^We completely acknowledge that a dichotomous measure of gender does not captures an individual’s gender identity nor all biological sexes. In our recent and ongoing research, we assessed gender differently.

^2^We ran the same analysis (PROCESS model 14) in the leader subsample (*n* = 148). The pattern of results remained the same and the interaction term team*prototypicality remained significant (*p* = 0.038) as was the index of moderated mediation (index = 0.21, *CI*95%: 0.04, 0.39).

^3^We also ran the analyses without control variables. Thereby, the pattern of results remained the same and only differed in its magnitude.

^4^We verified this finding using two analytical techniques: When authentic leadership was entered as predictor in the regression model, the interaction term no longer was a significant predictor. This pattern also applied when employing a covariance approach (SEM model): the interaction (joint effect) term only predicted trust in leader and job satisfaction when mediated by authentic leadership (and then trust in leader). For parsimony reasons, we only report the latter in the paper.
