# Supplementary material for: I am one of you! Team prototypicality as a facilitator for female leaders
Source: Front Psychol. 2022 Oct 20;13:859577. doi: 10.3389/fpsyg.2022.859577 (PMC9632852; doi:10.3389/fpsyg.2022.859577)
Supplement: Supplementary file 1 [file Data_Sheet_1.PDF]

Supplemental material

A.1 Table containing the variables and constructs assessed in Study 1

**construct/variable**	**items**	**answer format**
biological sex	Please indicate your gender.	• female<br>• male
age	Please indicate your age in years (e.g. 36).	Open answer format
nationality	Please indicate your nationality.	Open answer format
ethnic background	Please indicate your ethnic background.	• caucasian / white<br>• african-american / black<br>• hispanic / latino<br>• asian / pacific islands<br>• native americans
education	Which is your highest educational level?	• No school-leaving certificate<br>• Intermediate school-leaving certificate (9-10 years of schooling) / Obligatory schooling (elementary or secondary level)/mandatory school-leavin<br>• certificate<br>• Apprenticeship examination<br>• Qualification for university entrance<br>• Vocational baccalaureat<br>• Qualification for university of applied sciences<br>• University of applied sciences degree<br>• University degree (Bachleor)<br>• University degree (Master)<br>• University degree (PhD)
relationship status	What is your actual marital status?	• single<br>• in a relationship<br>• married<br>• divorced<br>• widowed
children	Do you have children?	• yes<br>• no
employment status	Please indicate which of the following applies to you.	• employed<br>• unemployed<br>• student full-time<br>• studying and working<br>• something different
working hours	I am…	• working full-time<br>• working part-time<br>• self-employed or project-based<br>• no permanent employment at the moment

contract	What kind of work contract do you have?	• Limited<br>• unlimited
sector	I am working in the...	• …public sector<br>• …private sector
leading responsibilities	Do you hold a leadership position?	• yes<br>• no
job title	Which of the following terms describes your job title best?	• clerk<br>• team leader<br>• department manager<br>• division manager<br>• member of executive board<br>• self-employed with employees
authentic leadership (ALQ, Walumbwa et al., 2008)	The following survey items refer to your managerial style, as you perceive it. Please judge how frequently each statement fits to you in a leading position.<br><br>As a leader I...<br>...say exactly what I mean.<br>...admit mistakes when they are made.<br>...encourage everyone to speak their mind.<br>...tell you the hard truth.<br>...display emotions exactly in line with feelings.<br>...demonstrate beliefs that are consistent with actions.<br>...make decisions based on my core values.<br>...ask you to take positions that support your core values.<br>...make difficult decisions based on high standards of ethical conduct.<br>...solicit views that challenge my deeply held positions.<br>...analyze relevant data before coming to a decision.<br>...listen carefully to different points of view before coming to conclusions.<br>...seek feedback to improve interactions with others.<br>...accurately describe how others view my capabilities.<br>...know when it is time to reevaluate my position on important issues.<br>...show I understand how specific actions impact others.	• not at all<br>• once in a while<br>• sometimes<br>• fairly often<br>• frequently
team prototypicality (adopted from	According to these results and information, I believe that...	• 1 = do not agree at all to 7 = fully agree

Ullrich et al., 2009)	• I represent what is characteristic for my team.<br>• I represent what my team has in common.	
Organizational identification (Kreiner & Ashforth, 2004)	Please indicate your agreement with the following statements by selecting the option which comes closest to your opinion.<br>Please still imagine you are part of the organization mentioned in the previous scenarios, being employed there for a while. You have the possibility to choose on a scale from "strongly disagree" to "strongly agree".<br>• When I talk about this organization, I usually say 'we' rather than 'they'.<br>• This organization's successes are my successes.<br>• When someone praises this organization it feels like a personal compliment.<br>• I have mixed feelings about my affiliation with this organization.<br>• I feel conflicted about being part of this organization.<br>• I have contradictory feelings about this organization.<br>• I don't have many feelings about this organization at all.<br>• I give little thought to the concerns of this organization.<br>• This organization doesn't have much personal meaning to me.<br>• This organization does shameful things.<br>• Sometimes, I find this organization to be disgraceful.<br>• I want people to know that I disagree with how this organization behaves.	• 1 = strongly disagree to 5 = strongly agree

Notes. None of the data has been used in any other publication.

A.2 Table containing the constructs assessed in Study 2

**construct/variable**	**items**	**answer format**
biological sex	Please indicate your gender.	• female<br>• male
age	Please indicate your age in years (e.g. 36).	Open answer format
nationality	Please indicate your nationality.	Open answer format

ethnic background	Please indicate your ethnic background.	• caucasian / white<br>• african-american / black<br>• hispanic / latino<br>• asian / pacific islands<br>• native americans
education	Which is your highest educational level?	• No school-leaving certificate<br>• Intermediate school-leaving certificate (9-10 years of schooling) / Obligatory schooling (elementary or secondary level)/mandatory school-leavin<br>• certificate<br>• Apprenticeship examination<br>• Qualification for university entrance<br>• Vocational baccalaureat<br>• Qualification for university of applied sciences<br>• University of applied sciences degree<br>• University degree (Bachleor)<br>• University degree (Master)<br>• University degree (PhD)
relationship status	What is your actual marital status?	• single<br>• in a relationship<br>• married<br>• divorced<br>• widowed
children	Do you have children?	• yes<br>• no
employment status	Please indicate which of the following applies to you.	• employed<br>• unemployed<br>• student full-time<br>• studying and working<br>• something different
working hours	I am…	• working full-time<br>• working part-time<br>• self-employed or project-based<br>• no permanent employment at the moment
contract	What kind of work contract do you have?	• Limited<br>• unlimited
sector	I am working in the...	• …public sector<br>• …private sector
leading responsibilities	Do you hold a leadership position?	• yes<br>• no
job title	Which of the following terms describes your job title best?	• clerk<br>• team leader<br>• department manager<br>• division manager

		• member of executive board<br>• self-employed with employees
gender of direct supervisor	Please indicate the gender of your direct supervisor / leader.	• female<br>• male
authentic leadership (ALQ, Walumbwa et al., 2008)	The following survey items refer to the managerial style of your supervisor / leader, as you perceive it. Please judge how<br>frequently each statement fits his or her leadership style.<br><br>My leader…<br>…says exactly what he or she means.<br>…admits mistakes when they are made.<br>…encourages everyone to speak their mind.<br>…tell you the hard truth.<br>…displays emotions exactly in line with feelings.<br>…demonstrates beliefs that are consistent with actions.<br>…makes decisions based on his or her core values.<br>…asks you to take positions that support your core values.<br>…makes difficult decisions based on high standards of ethical conduct.<br>…solicits views that challenge his or her deeply held positions.<br>…analyzes relevant data before coming to a decision.<br>…listens carefully to different points of view before coming to conclusions.<br>…seeks feedback to improve interactions with others.<br>…accurately describes how others view his or her capabilities.<br>…knows when it is time to reevaluate his or her positions on important issues.<br>…shows he or she understands how specific actions impact others.	• not at all<br>• once in a while<br>• sometimes<br>• fairly often<br>• frequently
organizational prototypicality (adopted from Ullrich et al., 2009)	Please indicate on the continuum from strongly disagree to strongly agree how much the following statements apply to your supervisor / leader. They focus on how representative is your supervisor / leader for the organization.	• 1 = strongly disagree to 5 = strongly agree

	• He or she represents what is characteristic about our organization.<br>• He or she is a good example of the kind of people that are in the organization.<br>• He or she stands for what people who work in my organization have in common.<br>• He or she is very similar to most people in my organization.	
team prototypicality (adopted from Ullrich et al., 2009)	Please indicate on the continuum from strongly disagree to strongly agree how much the following statements apply to your supervisor / leader. They focus on how representative is your supervisor / leader for the team you are working in.<br>• He or she represents what is characteristic for my team.<br>• He or she is a good example of the kind of people that are in my team.<br>• He or she stands for what people who work in my team have in common.<br>• He or she is very similar to most people in my team.	• 1 = strongly disagree to 5 = strongly agree
affective motivation to lead (Chan & Drasgow, 2003)	Now, it is about you. Please indicate on the continuum from strongly disgaree to strongly agree how much the following statements apply to you.<br>• Most of the time, I prefer being a leader rather than a follower when working in a group.<br>• I am the type of person who is not interested to lead others.<br>• I am definitely not a leader by nature.<br>• I am the type of person who likes to be in charge of others.<br>• I believe I can contribute more to a group if I am a follower rather than a leader.<br>• I usually want to be the leader in the groups that I work in.<br>• I am the type who would actively support a leader but prefers not to be appointed as leader.	• 1 = strongly disagree to 5 = strongly agree

	• I have a tendency to take charge in most groups or teams that I work in.<br>• I am seldom reluctant to be the leader of a group.	
Organizational identification (Kreiner & Ashforth, 2004)	Please indicate on the continuum from strongly disagree to strongly agree how much the following statements apply to you.<br>They refer to the organization you are working in.<br>• When I talk about this organization, I usually say ‘we’ rather than ‘they’.<br>• This organization’s successes are my successes.<br>• When someone praises this organization it feels like a personal compliment.<br>• I have mixed feelings about my affiliation with this organization.<br>• I feel conflicted about being part of this organization.<br>• I have contradictory feelings about this organization.<br>• I don’t have many feelings about this organization at all.<br>• I give little thought to the concerns of this organization.<br>• This organization doesn’t have much personal meaning to me.<br>• This organization does shameful things.<br>• Sometimes, I find this organization to be disgraceful.<br>• I want people to know that I disagree with how this organization behaves.	• 1 = strongly disagree to 5 = strongly agree
Trust & loyalty towards the leader (Podsakoff et al., 1990)	Please indicate on the continuum from strongly disgaree to strongly agree how much the following statements apply to you.<br>• I feel quite confident that my supervisor / leader will allways treat me fairly.<br>• My supervisor / leader would never try to gain advantage by deceiving workers.<br>• I have complete faith in the integrity of my supervisor / leader.	• 1 = strongly disagree to 5 = strongly agree

	• I feel a strong loyalty to my supervisor / leader.<br>• I would support my supervisor / leader in almost any emergency.<br>• I have a divided sense of loyalty toward my supervisor / leader.	
Job satisfaction (Spector, 1985)	Please indicate how you feel about different aspect of your work on the continuum from very dissatisfied to very satisfied.<br>• salary<br>• leader<br>• colleagues<br>• work itself<br>• overall	• 1 = very dissatisfied to 5 = very satisfied

Notes. None of the data has been used in any other publication.
